# Edward Drinker Cope

**Published:** 1897-05

**Authors:** 


### EDWARD DRINKER COPE.

It is rare that the world of scientific thought is called upon to mourn the loss of one more distinguished than Professor Cope. His work, through a life of constant labor, has made his name an authority throughout the domain of original investigation.

It is not a common experience, at least in this country, to find men willing to set aside practically all the amenities of life in order to advance knowledge in any line of work, but this is exactly what Edward D. Cope did. Born to wealth, he used it in the advancement of science, spending a fortune in his investigations.

His collections have been of the most varied and extensive character; indeed, it may be doubted whether there is another single individual in the United States who has secured and prepared a larger number of specimens of extinct and living species.

His writings will be his best monument, for they represent the labors of a life, and will be regarded as equal authority with the names "not born to die" in scientific annals.

While not connected with the dental profession, his work was very intimately associated with an important part of its study, the comparative anatomy of the teeth, and he was always ready to meet in dental societies and take part in the discussions where the subject was one in which he was particularly interested.

His death will leave a vacancy difficult to fill, but the influence of his self-sacrifice to science will, it is hoped, be an incentive to the few to continue the labor with the broad and comprehensive spirit which always characterized his investigations and deductions.

